# Therapeutic effect of chinese herbal medicine gu-ben-hua-shi (AESS) formula on atopic dermatitis through regulation of yes-associated protein

**DOI:** 10.3389/fphar.2022.929580

**Published:** 2022-10-12

**Authors:** Jinjing Jia, Luyao Feng, Siqi Ye, Ruiyue Ping, Xiumei Mo, Yu Zhang, Xiong Li, Dacan Chen

**Affiliations:** ^1^ State Key Laboratory of Dampness Syndrome of Chinese Medicine, the Second Affiliated Hospital of Guangzhou University of Chinese Medicine, Guangzhou, China; ^2^ Department of Dermatology, the Second Affiliated Hospital of Guangzhou University of Chinese Medicine, Guangzhou, China; ^3^ Guangdong-Hong Kong-Macau Joint Lab on Chinese Medicine and Immune Disease Research, Guangzhou University of Chinese Medicine, Guangzhou, China; ^4^ School of Traditional Chinese Medicine, Shenyang Pharmaceutical University, Shenyang, China

**Keywords:** atopic dermatitis, yes-associated protein, traditional Chinese medicine, Chinese herbal medicine, NF-κB signaling pathway

## Abstract

**Background:** Atopic dermatitis (AD) is a chronic and recurrent skin disease. At present, there is a lack of sufficiently effective and safe medicines that can be used for a prolonged time and reduce the recurrence of AD. The Gu-Ben-Hua-Shi (AESS) formula has been used for many years with a good clinical effect on AD but its specific treatment mechanism is unknown.

**Methods:** The main components of AESS were analyzed using ultra-high performance liquid chromatography (UPLC). The composition of AESS compounds in the serum from rats was analyzed using ultra-high performance liquid chromatography-mass spectrometry. An AD mouse model was constructed using 2,4-dinitrofluorobenzene stimulation in Balb/C mice and the effect on the reduction of skin lesions and Th1/Th2/Th17/Treg balance after AESS administration were measured. The effects of AESS serum on the proliferation and apoptosis of keratinocyte cell line HaCaT and adhesion of HaCaT to human monocyte cell line THP-1 were detected in an IFN-γ/TNF-α stimulated AD-like inflammatory cell model. The effects of Yes-associated protein (YAP) expression on the therapeutic effect and a related signaling pathway were also investigated.

**Results:** In total, 10 components were confirmed using UPLC, namely five organic acids, three flavonoids, and two chromogenic ketones. Additionally, the similarity of the three batches of samples (S1–3) was above 0.98, indicating that the formula samples have good uniformity. These 10 compounds were also detected in rat serum, suggesting that they are absorbed into rat blood as prototype components. Furthermore, AESS effectively reduced the skin lesions in the AD mouse model, regulated the Th1/Th2/Th17/Treg imbalance, improved the proliferation ability of the AD-like cell model, and inhibited HaCaT apoptosis and adhesion to THP-1 cells. It also reduced the expression of YAP in Th17 and Treg cells of the mouse spleen and increased YAP expression in the skin. The change in YAP expression in keratinocytes weakened the curative effect of AESS, and AESS exerted its effects through the NF-κB signaling pathway.

**Conclusion:** AESS may play a role in the treatment of AD by affecting the expression of YAP. These findings can be used to promote its use as an alternative medication for prolonged use with fewer side effects.

## Introduction

Atopic dermatitis (AD), also known as atopic eczema (Kantor et al., 2016), is a chronic, recurrent, and itchy skin disease, which affects a wide range of people: approximately 15% of children and 3% of adults (Kim et al., 2016). Its exact pathogenesis is unclear. In the past, it was thought that a Th2-dominated immune response played a key role in the pathogenesis of AD, causing Th1/Th2 imbalance and aggravating barrier dysfunction, which results in a vicious circle (Lloyd and Snelgrove, 2018). Recent studies have shown that the number of and functional differences in Th17 and Treg cells in the acute and chronic stages of AD play an important role in its pathogenesis (Lee et al., 2012; Noda et al., 2015), forming a complex and large Th1/Th2/Th17/Treg immune regulatory network. The Yes-associated protein (YAP) is a key downstream member of the Hippo signaling pathway (Moroishi et al., 2015). A previous study has shown that YAP plays an important role in the pathogenesis of AD by regulating the Th17/Treg balance (Jia et al., 2021).

At present, for the treatment of mild to moderate AD, modern medicine mainly uses topical glucocorticoids, calcineurin inhibitors, and emollients but these still cannot prevent flare-ups and there are adverse reactions from the long-term use of glucocorticoids and calcineurin inhibitors. Consequently, many patients doubly suffer from the disease and side effects of the drugs. For severe AD, the efficacy of biological agents, which is not as good as that for psoriasis, is heterogeneous, and studies on the side effects of long-term treatment are still insufficient (de Bruin-Weller et al., 2018). The role of traditional Chinese medicine (TCM) in the treatment of AD has been more internationally recognized. Many guidelines on AD refer to TCM therapy (Wollenberg et al., 2018), which is considered an alternative method for dealing with the treatment dilemma. In TCM, AD belongs to the category of “Four Bend Wind” (Yan et al., 2020). The Gu-Ben-Hua-Shi (abbreviated as AESS) formula was proposed by the leading Chinese medicine talent, Qihuang scholar Dr. Dacan Chen, and has proved clinically effective for many years in the treatment of AD (Wu et al., 2014; Liu and Mo, 2019; Jia et al., 2022) and obtained a national invention patent in China (No. ZL202110958138.2). However, its specific mechanism in AD is unknown. The aim of this study was to clarify its effect on YAP expression and therapeutic mechanism on AD.

## Materials and methods

### Preparation of AESS

Three batches (S1–3) of the prescribed herbs were purchased from different Chinese herbal medicine markets in several provinces of China (Supplemental Table S1). The plant names in AESS, checked with http://www.theplantlist.org, are shown in Table 1. Prim-*O*-glucosylcimifugin (4), Kaempferol-3-*O-*rutinoside (6), and 5-*O*-methylvisamitol glycoside 9) were purchased from Beijing Jinming Biotechnology Co., Ltd (Beijing, China). Isochlorogenic acid C (**10**) and narcissoside (**8**) were purchased from Chengdu Push Bio-Technology Co., Ltd (Chengdu, China). Rutin (**5**), chlorogenic acid (**2**), cryptochlorogenic acid **3**), and neochlorogenic acid (**1**) were purchased from Chengdu Must Biotechnology Co., Ltd (Chengdu, China). Isochlorogenic acid A (**7**) was purchased from Chengdu Desit Biotechnology Co., Ltd (Chengdu, China). The standard substances were accurately weighed to prepare a standard mother liquor at a final concentration of 1 mg/ml. Then, each standard mother liquor was diluted approximately 200 times with 40% MeOH to produce a 5 μg/ml solution. After centrifugation, the solution was filtered through a 0.22-μm filter, reserving the supernatant and storing it at 4 °C. For the single herb solution, 50 g of each medicinal herb was crushed through a 40- or 60-mesh sieve and the weighed powder underwent ultrasonic treatment for 30 min with an 80% methanol-water solution. For three batches of the formula extract preparation, the described herbal materials were extracted using the traditional decoction boiling method, with 10 times (g/ml) water and boiling for 1 h. The decoction was filtered and concentrated under reduced pressure at 60°C and freeze-dried. Subsequently, 30 mg of the sample powder was accurately weighed, 80% MeOH was added to completely dissolve it, and ultrasonic treatment was conducted for 30 min. Next, ultra-pure water was added to prepare a sample solution with a concentration of 10 mg/ml and a solvent of 40% MeOH. The solution was filtered through a 0.22-μm filter membrane and placed at 4°C for later use.

**TABLE 1:** Herbal composion information of Gu-Ben-Hua-Shi (AESS).

Chinese name	English name	Accepted scientific name	Dose (/g)
fangfeng	Divaricate Saposhniovia Root	*Saposhnikovia divaricata* (Turcz.) Schischk	10
ezhu	Zedoary	*Curcuma kwangsiensis* S.G.Lee and C.F.Liang	10
jinyinhua	Honeysuckle Flower	*Lonicera japonica* Thunb	10
shengdihuang	Rehmannia Root	*Rehmannia glutinosa* (Gaertn.) DC.	10
huaihua	Sophorae Flos	*Sophora jaubertii* Spach	10
baizhu	Atractylodis Macrocephalae Rhizoma	*Atractylodes macrocephala* Koidz	15
yiyiren	Coicis Semen	*Coix lacryma-jobi* var. *ma-yuen* (Rom.Caill.) Stapf	20
cangzhu	Swordlike Atractylodes Rhizome	*Atractylodes lancea* (Thunb.) DC.	10

### Ultra-high performance liquid chromatography (UPLC) conditions

UPLC analysis was performed on a Waters Acquity UPLC system (Waters, USA) equipped with a binary solvent delivery pump, an autosampler, and a photodiode array detector. The Acquity UPLC HSS T3 (2.1 × 150 mm, 1.8 μm) column (Waters, Ireland) was used. Chromatographic methanol and acetonitrile were produced by Merck (Darmstadt, Germany), and formic acid was produced by Kermeo Chemical Reagent Co., Ltd. (Tianjin, China). The chromatographic run was conducted with a flow of 0.30 ml/min and the temperature of the column was maintained at 40 °C with an injection volume of 5 μL. The detection wavelength was set at 300 nm. The mobile phase system is presented in Supplemental Table S2.

The sample solutions (S1–S3) were analyzed according to the above conditions. Chromatographic fingerprints were generated for the three batches of samples. A reference standard chromatographic fingerprint was generated using Similarity Evaluation System for Chromatographic Fingerprint of TCM (version 2012). The same sample solution (e.g., S1) was sampled six consecutive times using rutin (5) as the reference peak. Then, the same sample solution was analyzed at 0, 2, 4, 8, 12, 24, 36, 48, and 72 h. The same batch of samples was taken and six samples of the sample solution were prepared in parallel. The samples were sequentially analyzed according to the above chromatographic conditions.

### Establishment of the AD mouse model

The experimental animal ethics committee of the Guangdong Provincial Hospital of Chinese Medicine approved all experiments. Female Balb/C mice were randomly divided into four groups: the normal control group (Ctrl), AD model group (AD), AD + AESS group (AESS), and AD + dexamethasone group (DEX), with six mice in each group. The backs of the mice were shaved and sensitized using 150 μL of 0.15% 2,4-dinitrofluorobenzene (DNFB; Sigma, USA; dissolved in acetone and olive oil at 3:1 as a matrix solution) on the dorsal back and 20 μL on the ears and alternately stimulated with 0.15% DNFB twice a week (Han et al., 2018). The mice in the Ctrl and AD groups, AESS group, and DEX group were intragastrically administered daily with normal saline, a crude drug dose of 14.27 g/kg AESS formula (according to the conversion of human clinical dosage; Zhao and Sun, 2010), and 1 mg/kg dexamethasone (Sigma, United States; Lee et al., 2019), respectively. The dermatitis score was calculated from the sum of the four indicators (Kim et al., 2013): erythema/bleeding, eschar/dryness, *epidermis* exfoliation/erosion, and edema. The severity of each was calculated using 0–3 points, where 0 = none, 1 = light, 2 = medium, and 3 = heavy.

### Determination of the Th1/Th2/Th17/Treg ratio in mouse spleen cells

The mononuclear cells were isolated from the spleen of the mice. The proportion of each subtype was detected usingNovocyte D2060R flow cytometry (Agilent Technologies, United States). The CD3^+^CD4+IFN-γ+ cells represented Th1 cells, CD3^+^CD4+IL-4+ cells represented Th2 cells, CD3^+^CD4+IL-17 + cells represented Th17 cells, and CD3^+^CD4^+^CD25+Foxp3+ cells represented Treg cells.

### Determination of the histopathological and immunohistochemical results

All skin tissue samples were fixed in 4% paraformaldehyde and embedded in paraffin for subsequent histopathological and immunohistochemical staining. Hematoxylin and eosin staining and immunohistochemical staining followed the standard procedure (Pinheiro et al., 2008). Skin thickness was measured using Image-Pro Plus 6.0 (Media Cybernetics, United States). The positive expression of immunohistochemistry was observed as yellow or brown granules in the cytoplasm and/or nucleus. Ten fields were randomly selected under ×400 magnification to observe the intensity of the cell staining and calculate the percentage of positive cells. The positive rate <5% was 0, 5–25% was 1, 25–50% was 2, 50–75% was 3, and >75% was four; according to the staining intensity, it was divided into four grades: no staining was 0, light yellow was 1, yellow-brown was 2, and brown was 3. The percentage of positive cells in each visual field multiplied by the staining intensity was the score of the visual field and the final score was the average of the five visual field scores. For the score: 0 points were negative (-), 1–4 points were weakly positive (++), 5–8 points were moderately positive (++), and 9–12 points were strongly positive (++ +).

### Induction and differentiation of the T cell subsets

The spleen mononuclear cells were separated using the Mouse Naive CD4^+^ T Cell Isolation Kit (Stemcell Technologies, Canada), and then the differentiation of naive CD4^+^ T cells into Th17 and Treg cells was induced by the Th17 and Treg cell differentiation kits (R&D systems CellXVivo, USA), respectively.

### Real-time quantitative PCR (qRT-PCR) was used to detect the expression of YAP mRNA

Total RNA was extracted from different T cell subsets (Th17 and Treg cells), mouse skin lesions, and HaCaT cells using the TRIzol method. Complementary DNA (cDNA) was synthesized using reverse transcription. An Exicycler 96 Real time PCR instrument (BIONEER, Korea) was used to measure mRNA expression, with GAPDH and β-actin used as the internal reference. Primer sequences are shown in Supplemental Table S3.

### Western blotting for the detection of the expression of YAP

Total protein was extracted from the skin lesions of the mice. The expression of YAP was detected using sodium dodecyl sulfate-polyacrylamide gel electrophoresis, membrane transfer, antibody incubation, and western blotting. The primary antibodies used included those against: YAP, 1:1,000, Cell Signaling Technology, United States, #14074; IκB-α, 1:500, Wanleibio Co., Ltd. China, WL01936; p-IκB-α, 1:500, Wanleibio Co., Ltd. WL02495; NF-κB p65, 1:500, Wanleibio Co., Ltd. WL01273b; p-NF-κB p65, 1:500, Wanleibio Co., Ltd. WL02169; and β-actin, 1:10,000, Santa Cruz Biotechnology, United States, sc-47778.

### Preparation of the AESS serum formula in rats

Male Sprague Dawley rats weighing 220–250 g were randomly divided into the control group and AESS group. The rats were fasted for 8–12 h before gastric administration. The AESS group was administered a crude drug dose of 8.54 g/kg daily (according to the conversion of human clinical dosage; Huang et al., 2004) AESS formula by gastric administration twice a day for 5 days and the control group was administered an equal volume of physiological saline. Sixty minutes after the final administration, blood was collected and the serum was separated. A 0.22-μM filter was used for sterilization and the serum was stored at -80 °C (Wei et al., 2016; Han et al., 2017).

### Analysis of AESS compounds in the rat serum by ultra-high performance liquid chromatography-mass spectrometry

The composition of AESS compounds in the serum of rats was analyzed using UPLC-MS/MS. A Triple-TOF 6500 + Mass Spectrometer (AB SCIEX, United States) combined with a UPLC system (Shimadzu, Japan) was used. The Hypersil GOLD (100 × 2.1 mm, 1.9 μm; Thermo Fisher Scientific) column was used. Chromatographic methanol and acetonitrile were produced by Merck, and formic acid was produced by Kermeo Chemical Reagent Co., Ltd. The chromatographic run was conducted with a flow of 0.30 ml/min and the temperature of the column was maintained at 40°C with an injection volume of 5 μl. The mobile phase system is presented in Supplemental Table S4. The operating parameters of MS/MS were set as follows: scan range, m/z 100–1,500; electrospray ionization source, positive or negative mode; capillary temperature, 320 °C; positive mode, 5500 V of spray voltage; negative mode, 4500 V of spray voltage; and scan mode, multi reaction mooitoring (MRM). The declustering potential and collision energy of compounds are presented in Supplemental Table S5.

### Cell culture

The human immortalized keratinocyte line, HaCaT, was obtained from Shanghai Xiangf Biotechnology Co., Ltd. And cultured in Dulbecco’s modified Eagle medium (DMEM; Gibco/Thermo Fisher Scientific, United States) at 37°C and 5% CO_2_. The YAP inhibitor verteporfin (VP, 2 μl; Shanghai Yuanye Biotechnology Co., Ltd., China; Morice et al., 2020) was added to the cell culture medium to inhibit YAP expression in HaCaT cells.

### Establishment of an AD-like inflammatory cell model

The HaCaT cells in the logarithmic growth phase were treated with a mixture of serum, IFN-γ, and TNF-α (Sino Biological, China) to create an AD-like inflammatory cell model (Savinko et al., 2012; Hou et al., 2017). The control group was treated with a mixture of normal rat serum and solvent and the AD group was treated with a mixture of normal rat serum and 10 ng/ml IFN-γ + 10 ng/ml TNF-α. The AESS group was treated with a mixture of AESS serum and 10 ng/ml IFN-γ + 10 ng/ml TNF-α and the DEX group was treated with a mixture of 20 μg/ml dexamethasone (Xie et al., 2020) and 10 ng/ml IFN-γ + 10 ng/ml TNF-α.

### Cell proliferation assay

The proliferation of cells was detected using MTT assays. HaCaT cells were inoculated in 96-well plates. Each well contained 5 × 10^3^ cells/200 μL culture medium. After 24, 48, and 72 h, 20 μL of MTT was added to each well. After 4 h of incubation, the supernatant was discarded. Then, 150 μL of DMSO was added to each well and the crystal was fully dissolved for 10 min. The absorbance was determined at 570 nm using a microplate reader (BioTek, United States).

### Apoptosis assay

The cells were inoculated in 6-well plates for 24 h. Then, the cells were digested and collected using an apoptosis detection kit (Wanlei Biotech Co., Ltd.). Subsequently, 500 μl of binding buffer was added to gently suspend the cells, 5 μL of annexin V-FITC was added, and 5 μl of PI staining solution was added. The cells were then incubated at room temperature for 15 min in the dark for flow cytometry.

### Cell adhesion assay

The HaCaT cells (7.0 × 10^4^ cells/well) were seeded into 12-well plates and treated for 24 h according to the experimental group. The THP-1 cells (7.0 × 10^5^ cells/well) were incubated in RPMI medium (Gibco) containing 2% fetal bovine serum and 10 μg/ml 2ʹ,7ʹ-bis-(2-carboxyethyl)-5-(and-6)-carboxyfluorescein acetoxymethyl ester (BCECF/AM; Beyotime, China) for 30 min. The fluorescently labeled THP-1 cells were collected and resuspended with DMEM. Then, the fluorescence-labeled THP-1 cell suspension was co-cultured with the HaCaT cells at 37°C for 30 min. Next, the cells were washed with phosphate-buffered saline twice to remove the no-adherent cells. The number of adherent THP-1 cells was counted using a fluorescence microscope (100×; Lee et al., 2019).

### Statistical analysis

SPSS 19.0 was used for statistical analysis. Data are expressed as mean ± standard error of the mean. A chi-square test was used to compare the positive rate of immunohistochemical staining among groups and a Mann–Whitney *U* test was used to compare the expression intensity grade. A *t*-test was used to compare two groups. Additionally, an ANOVA was used to compare three groups or more, followed by the post-hoc LSD test for equal variance and Dunnett’s T3 for unequal variance. A significance level of *p* < 0.05 indicates that the difference is significant.

## Results

### Component identification of the AESS formula

**FIGURE 1:**
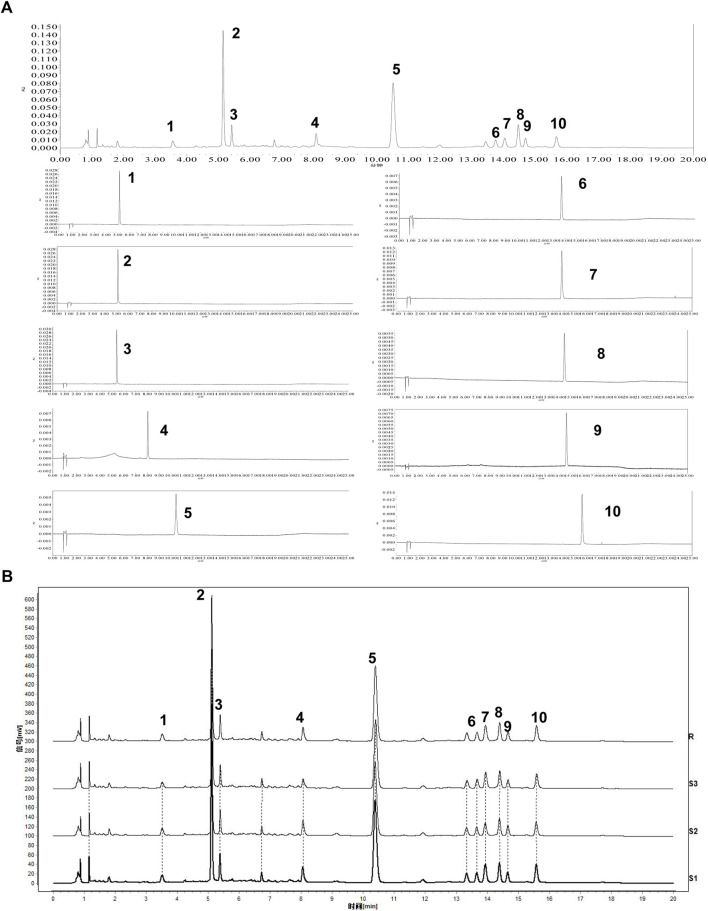
Analysis of the Main Components of AESS formula. **(A)** Typical UPLC chromatogram of the sample and 10 reference standards (1: Neochlorogenic acid, 2: Chlorogenic acid, 3: Cryptochlorogenic acid, 4: Prim-*O*-glucosylcimifugin, 5: Rutin, 6: Kaempferol-3-*O*-Rutinoside, 7: Isochlorogenic acid A, 8: Narcissoside, 9: 5-*O*-methylvisamitol glycoside. 10: Isochlorogenic acid C) **(B)** UPLC chromatographic fingerprints of three samples (S1-S3) and simulative median chromatogram (R).

It was calculated that the total 95g crude drug yielded 23.142g lyophilized powder. In total, 10 components were confirmed by comparison with those of the standards, namely five organic acids, three flavonoids, and two chromogenic ketones (Figure 1A).

### UPLC fingerprint analysis

The relative retention time and peak area relative standard deviation (RSD) of 10 common chromatographic peaks were less than 2.0%, indicating good precision of the instrument. Additionally, the relative retention time and peak area RSD of each chromatographic peak were less than 6.0%, indicating that the tested solution had good stability within 72 h and that the method had good repeatability. The similarity of the three batches of samples was above 0.98, indicating that the formula samples have good uniformity (Figure 1B).

### Quantitative analysis of the main components

Since the content of the component 1 (Neochlorogenic acid) was very low, and the content of the other nine components was high and stable, this paper only took these nine components (2–10) for subsequent quantitative analysis. The linear regression was conducted with the peak area as the Y coordinate and concentration as the X coordinate, and the regression equation was obtained. The limit of detection and limit of quantification were determined at a signal-to-noise ratio of approximately 3 and 10, respectively. The results are shown in Table 2. For precision, repeatability, and stability, the RSDs were less than 1.8%, 5.6%, and 5.2%, respectively. The recovery of the method was in the range of 97.55%–102.64%, with an RSD of less than 8.20%, indicating that the established method could accurately determine the nine compounds. Three batches of the samples were determined, and the results are shown in Table 3. The content of eight of the compounds was above 1 mg/g and the RSD of the nine components was below 40%, indicating that the uniformity of the formula is controllable.

**TABLE 2:** Linear regression data, LOD and LOQ of nine determined analytes.

Peak	Regression equation	Liner range (μg/ml)	*r* ^2^	LOD (ng)	LOQ (ng)
2	y = 19,471.7175 x—59,918.8199	12.5–200	0.9995	0.25	1.00
3	y = 14,955.4017 x—23,149.9767	6.25–100	0.9996	1.25	5.00
4	y = 11,677.3871 x—4,840.1271	6.25–100	0.9999	0.25	1.00
5	y = 5,406.0547 x—18,690.1441	18.75–300	0.9993	1.25	5.00
6	y = 15,457.0902 x—33,668.3051	6.25–100	0.9991	0.25	1.25
7	y = 7,674.7793 x—7,251.4343	3.125–50	0.9998	7.50	30.00
8	y = 5,998.88847 x—7,848.63347	6.25–100	0.9991	1.25	2.60
9	y = 10,587.1051 x—4,031.1525	3.125–50	0.9997	0.25	1.25
10	y = 16,432.0912 x—67,596.8962	6.25–100	0.9992	7.50	30.00

**TABLE 3:** Quantitative determination of nine main compounds in three batches of AESS.

Peak	Content (μg/g, mean ± SD, n = 3)	RSD (%)
2	4,720.84 ± 10.54	22.32
3	1,115.31 ± 1.84	16.51
4	1,076.00 ± 3.83	35.62
5	19,915.91 ± 40.27	20.22
6	1,309.09 ± 2.41	18.42
7	1,249.35 ± 3.76	30.08
8	3,070.69 ± 5.65	18.39
9	900.70 ± 1.82	20.24
10	1,365.10 ± 3.60	26.36

Content: Average content of each compound in three batches.

RSD: Variation of content of each compound in three batches.

### Qualitative analysis of AESS in rats serum samples

**FIGURE 2:**
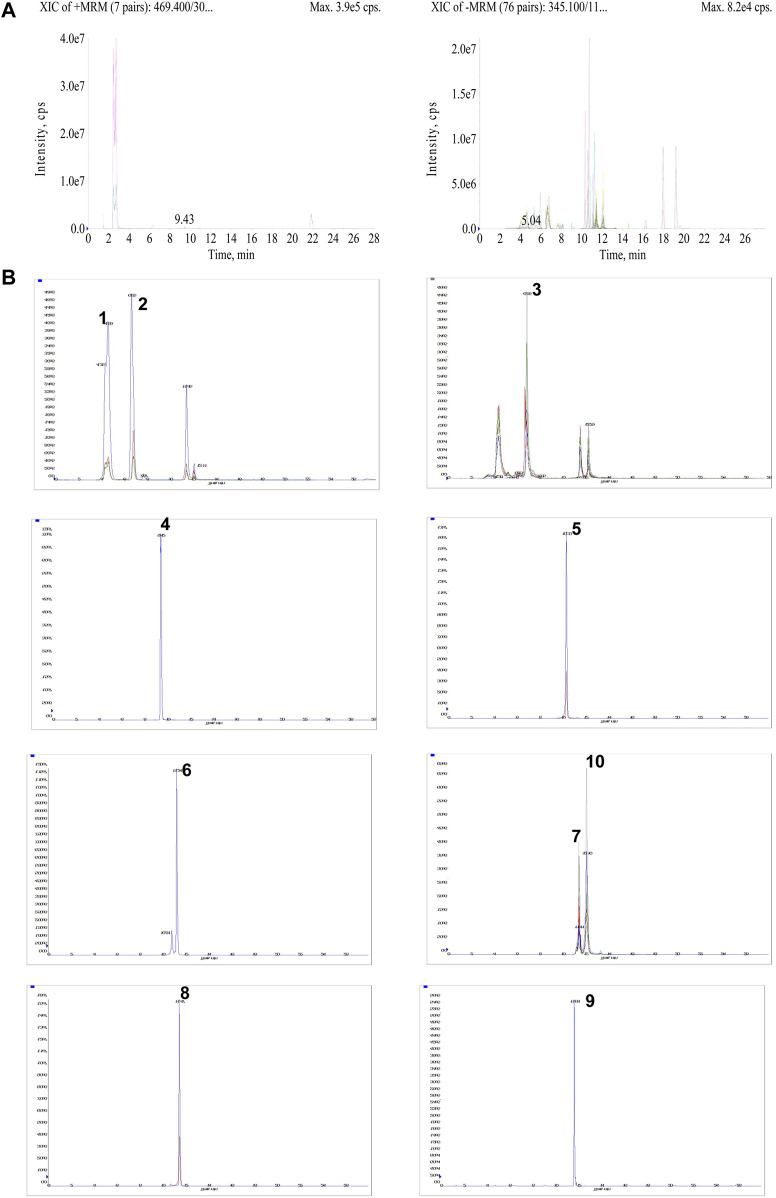
Qualitative analysis of AESS in rats serum samples. **(A)** UPLC-MS/MS chromatograms of drug-containing serum in positive and negative ion mode. **(B)** The extracted ion chromatograms of 10 compounds in AESS serum samples.

UPLC-MS technology was used for the rapid determination of components in the drug-containing serum of rats. The UPLC-MS/MS chromatograms of drug-containing serum in positive and negative ion mode are shown in Figure 2A and extracted ion chromatograms of 10 compounds in AESS serum samples are shown in Figure 2B. These 10 compounds were detected in serum, showing that they were absorbed into rat blood as prototype components. These results suggest that these 10 components underlie its therapeutic effect in AD.

### AESS reduces skin lesions in the AD mouse model

**FIGURE 3:**
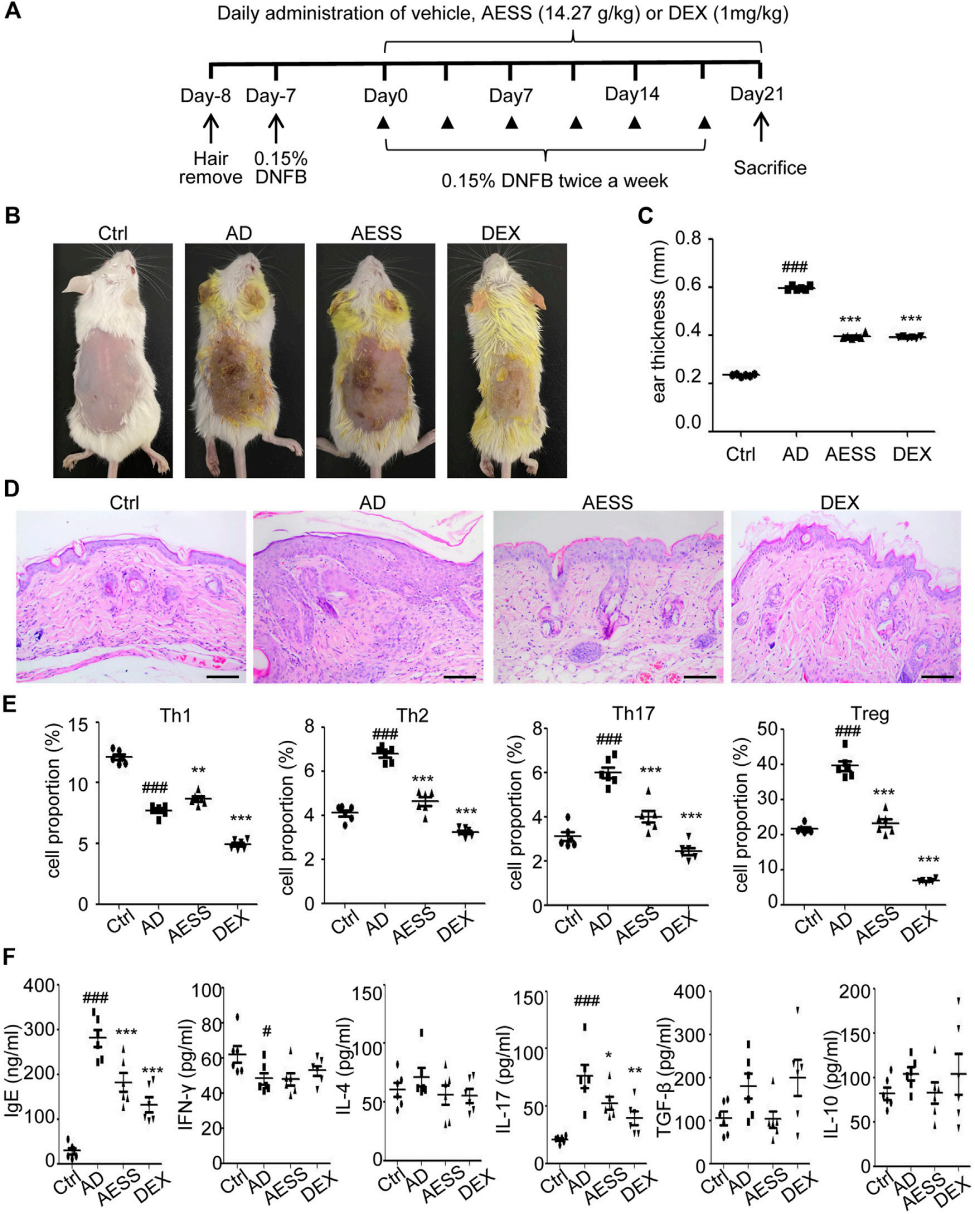
AESS formula could relieve skin lesions and regulate Th1/Th2/Th17/Treg balances of AD mice model **(A)** Experimental scheme for the DNFB-induced AD model. **(B)** General pictures of the mice. **(C)** Ear thickness of the mice (mm). **(D)** HE staining for histopathological features (bar length = 100 μm). **(E)** The proportion of Th1/Th2/Th17/Treg cells in the peripheral blood of mice was determined using FACS analysis. **(F)** Levels of IgE, IFN-γ, IL-4, IL-17A, TGF-β and IL-10 in the peripheral blood of mice. ELISA for three replicates. Number of animals = 6. Ctrl: Control mice; AD: Atopic dermatitis model mice; AESS: Mice intragastrically administered of 14.27 g/kg AESS formula; DEX: Mice intragastrically administered of 1 mg/kg dexamethasone. #*p* < 0.05, ##*p* < 0.01, ###*p* < 0.001 *v. s.* Ctrl; *p < 0.05, **p < 0.01, ***p < 0.001 *v. s.* AD.

The AD mice model was conducted using DNFB treatment (Figure 3A). The skin lesions and pathological and immunological characteristics of DNFB-sensitized AD mice were very similar to those of human AD (Figure 3B). After the intragastric administration of AESS, the symptoms of erythema and scales were alleviated, skin lesions became thinner, dermatitis score decreased, and degree of ear swelling was alleviated in the mice. However, these changes were more obvious in the group of the positive control drug dexamethasone (Figures 3B–D and Table 4 and 5).

**TABLE 4:** Dermatitis score of mice.

Group	Dermatitis score
Ctrl	0.000 ± 0.000
AD	6.000 ± 0.365^a^
AESS	3.167 ± 0.167^**^
DEX	1.500 ± 0.224^***^

^###^ *p* < 0.001 vs Ctrl.

^**^ *p* < 0.01.

^***^ *p* < 0.001 vs AD.

Ctrl: control mice; AD, atopic dermatitis model mice; AESS, mice intragastrically administered of 14.27 g/kg AESS, formula; DEX, mice intragastrically administered of 1 mg/kg dexamethasone.

**TABLE 5:** Thicknesses of the skin tissues of mice.

Group	Skin thickness (µm)
Ctrl	126.25 ± 7.59
AD	221.93 ± 3.24###
AESS	167.43 ± 6.89***
DEX	148.98 ± 10.91***

###p < 0.001 vs Ctrl.

***p < 0.001 vs AD.

Ctrl, Control mice; AD: atopic dermatitis model mice; AESS: Mice intragastrically administered of 14.27 g/kg AESS, formula; DEX: Mice intragastrically administered of 1 mg/kg dexamethasone.

### AESS increases the ratio of Th1 cells and decreases that of Th2, Th17, and Treg cells in the AD mouse model

Th1, Th2, Th17, and Treg cells were isolated from mouse spleens and detected using flow cytometry. The ratio of Th1 cells in the spleens of the AD group decreased, whereas that of Th2, Th17, and Treg cells increased. After 4 weeks of AESS administration, the ratio of Th1 cells increased and that of Th2, Th17, and Treg cells decreased. However, the ratio of Th1, Th2, Th17, and Treg cells decreased significantly after the intragastric administration of dexamethasone (Figure 3E).

### AESS reduces the expression of IgE and IL-17 in the serum of the AD mouse model

ELISA showed that the expression of IgE and IL-17 increased and that of IFN-γ decreased in the AD group. Therefore, AESS can reduce IgE and IL-17 levels in serum (Figure 3F).

### AESS reduces YAP expression in spleen Th17 and Treg cells in the AD mouse model

**FIGURE 4:**
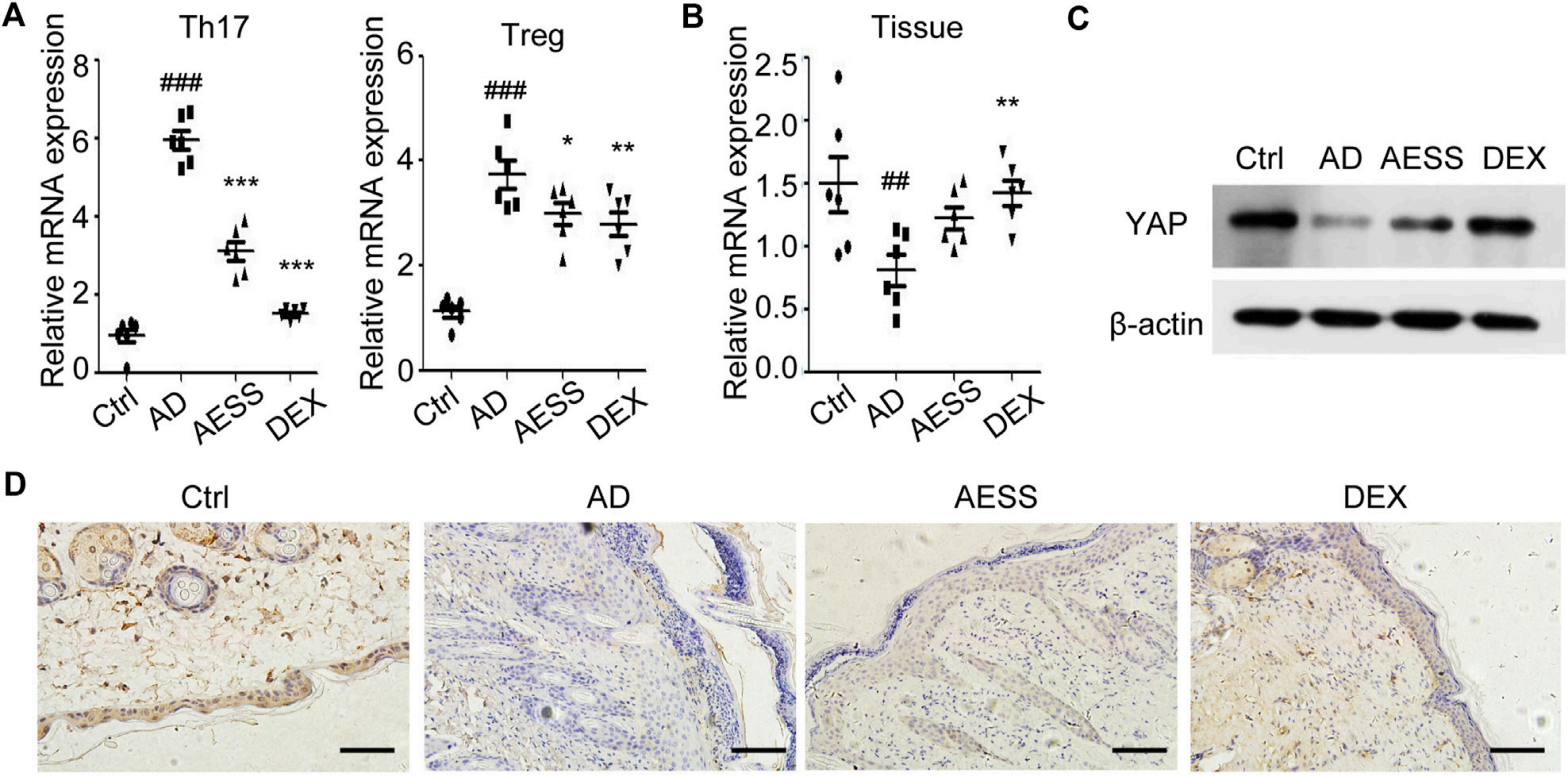
AESS formula could inhibit YAP expression in Th17 and Treg cells and induce YAP expression in epidermis of AD mice model. **(A)** Relative YAP protein expression in Th17/Treg cells from spleen mononuclear cells of mice. **(B)** Relative YAP mRNA expression in skin tissues of mice. **(C)** Relative YAP protein expression in skin tissues of mice. **(D)** Immunohistochemical analysis of YAP expression in the skin of mice. Scale bar = 100 μm qRT-PCR for three replicates. Number of animals = 6. Ctrl: Control mice; AD: Atopic dermatitis model mice; AESS: Mice intragastrically administered of 14.27 g/kg AESS formula; DEX: Mice intragastrically administered of 1 mg/kg dexamethasone. ##*p* < 0.01, ###*p* < 0.001 *v. s.* Ctrl; *p < 0.05, **p < 0.01, ***p < 0.001 *v. s.* AD.

Native CD4 + T cells were isolated from mice spleens and induced to differentiate into Th17 and Treg cells. The expression of YAP mRNA in Th17 and Treg cells was detected using qRT-PCR. YAP mRNA expression increased in Th17 and Treg cells in the AD group. After the intragastric administration of AESS, the expression of YAP mRNA in Th17 and Treg cells decreased, but not as significantly as that in the DEX group (Figure 4A).

### AESS increases the expression of YAP in the skin lesions of the AD mouse model

The expression of YAP mRNA and YAP protein in the mouse skin lesions was detected using qRT-PCR and western blotting, respectively. AESS slightly increased the expression of YAP in mouse skin lesions but not as obviously as dexamethasone (Figures 4B,C and Supplemental Figure S1A).

The results of immunohistochemistry showed that the positive rate of YAP was 83.33% (5/6) in the skin tissue of the control group and only 50.00% (3/6) in the skin lesions of the AD group, and the difference was significant (*χ* ^*2*^ = 5.333, *p* = 0.021). AESS slightly increased the positive rate of YAP expression in the skin lesions of mice but the difference was not significant. However, dexamethasone significantly increased the positive rate of YAP (*χ* ^*2*^ = 5.333, *p* = 0.021). The statistical analysis of immunohistochemical expression intensity grade data showed that there was no significant difference between the AD and AESS groups (*H* = 7.500, *p* = 0.058) but there was a significant difference between the AD and DEX groups (*H* = 4.000, *p* = 0.016; Table 6 and Figure 4D).

**TABLE 6:** The expression of YAP in mice skin lesions.

Group	Case	Expression grade				Positive rate (%)
		-	+	2+	3+	
Ctrl	6	1	1	1	3	83.33
AD	6	5	1	0	0	15.67^#^
AESS	6	2	1	3	0	66.67
DEX	6	1	1	3	1	83.33^*^

#*p* < 0.05 v. s. Ctrl.

*p < 0.05 v. s. AD.

Ctrl, Control mice; AD, atopic dermatitis model mice; AESS, Mice intragastrically administered of 14.27 g/kg AESS, formula; DEX, Mice intragastrically administered of 1 mg/kg dexamethasone.

### Determination of the AESS serum concentration

**FIGURE 5:**
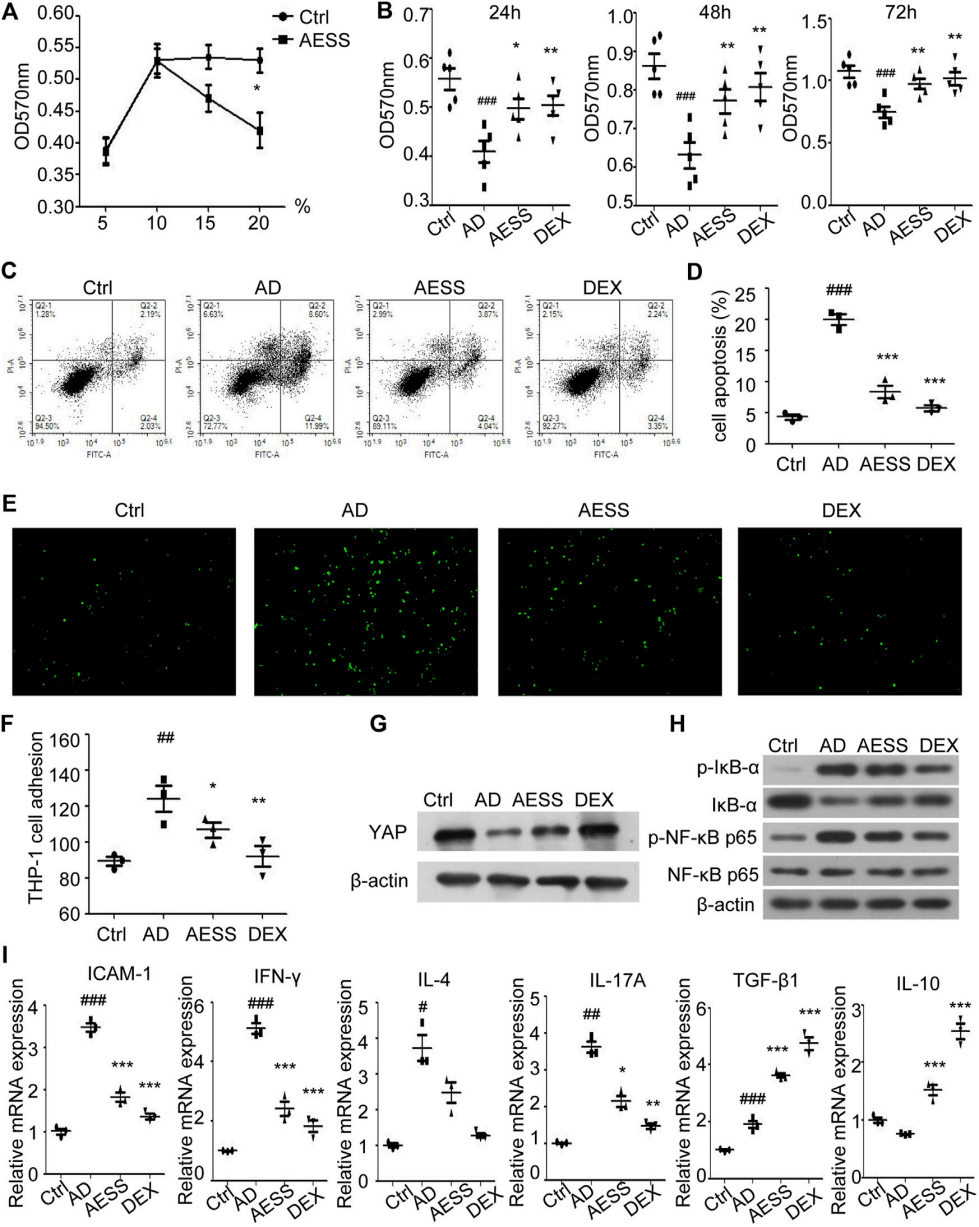
AESS contained serum can improve proliferation, reduce apoptosis of AD-like cell model and reduce adhesion rate to THP-1 cells. **(A)** Determination of AESS contained serum concentration in rats by MTT. **(B)** Cell proliferation was examined 24 h after culture with AESS contained serum by MTT **(C,D)** Apoptosis was examined 24 h after culture with AESS contained serum by FACS analysis. **(E,F)** Adhesion of AD-like cells to THP-1 cells 24 h after culture with AESS contained serum (100 ×). **(G)** YAP expression after culture with AESS contained serum. **(H)** IκB-α, p-IκB-α, NF-κB p65 and p-NF-κB p65 expression after culture with AESS contained serum. **(I)** ICAM-1 and inflammatory cytokines (IFN-γ, IL-4, IL-17A, TGF-β and IL-10) mRNA expression. MTT for five replicates. Cell apoptosis, adhesion assay and qRT-PCR for three replicates. Ctrl: Control HaCaT cells; AD: AD-like cells were established by HaCaT cells culture with 10 ng/ml TNF-α/IFN-γ; AESS: AD-like cells culture with AESS contained serum; DEX: AD-like cells culture with 20 μg/ml dexamethasone. #*p* < 0.05, ##*p* < 0.01, ###*p* < 0.001 *v. s.* Ctrl; *p < 0.05, **p < 0.01, ***p < 0.001 *v. s.* AD.

HaCaT cells in the logarithmic growth phase were treated with different concentrations (5%, 10%, 15%, and 20%) of normal rat serum and AESS serum. After 24 h of culture, the proliferation of HaCaT cells was detected using MTT assays. Compared with that in normal rat serum, the growth of the HaCaT cells treated with 15% and 20% AESS serum was significantly inhibited, indicating toxicity. Therefore, 10% medicated serum was selected as the follow-up experimental concentration (Figure 5A).

### AESS serum increases the proliferation of the AD-like cell model

MTT assays were used to detect the proliferation ability of the cells in each group. The proliferation level of the AD-like cell model made using a mixture of 10 ng/ml IFN-γ and TNF-α decreased at 24, 48, and 72 h. AESS serum increased the proliferation ability of the AD-like cell model but not as obviously as the increase by adding 20 μg/ml dexamethasone (Figure 5B).

### AESS serum reduces the apoptosis of the AD-like cell model

Annexin V-FITC/PI double staining flow cytometry was used to detect the apoptosis of cells in each group. The apoptosis level of the AD-like cell model increased, and the AESS serum reduced the apoptosis level of the AD-like cell model but not as obviously as the reduction by adding 20 μg/ml dexamethasone (Figures 5C,D).

### AESS serum reduces the adhesion of AD-like cells to THP-1 cells

The adhesion rate of HaCaT cells stimulated by 10 ng/ml IFN-γ and TNF-α to the human monocyte THP-1 was increased. AESS serum reduced the adhesion of HaCaT cells but not as obviously as the reduction by adding 20 μg/ml dexamethasone (Figures 5E,F).

### Effect of AESS serum on YAP expression and the NF-κB signaling pathway

Western blotting was used to detect the expression of YAP, IκB-α, p-IκB-α, NF-κB p65, and p-NF-κB p65. The expression of YAP in HaCaT cells stimulated by 10 ng/ml IFN-γ and TNF-α decreased, and AESS increased the expression of YAP (Figure 5G and Supplemental Figure S1B). The expression of total NF-κB p65 protein was unchanged, that of p-IκB-α and p-NF-κB proteins increased, and that of IκB-α decreased, indicating that the NF-κB signaling pathway is activated in response to AD. Furthermore, AESS serum reduced the expression of the p-IκB-α and p-NF-κB p65 proteins, indicating that AESS inhibits the activity of the NF-κB signaling pathway in the AD-like cell model (Figure 5H and Supplemental Figure S1C).

### AESS serum reduces intercellular cell adhesion molecule-1 and IL-17 mRNA levels in the AD-like cell model

ICAM-1 mRNA from cell lysates was upregulated in AD-like HaCaT cells and downregulated after AESS serum or dexamethasone treatment, indicating that the reduction of adhesion to monocytes occurred *via* the downregulation of ICAM-1 after AESS serum treatment (Figure 5I). However, although the trend of inflammatory cytokine IL-17A mRNA was the same, TGF-β and IL-10 mRNA was upregulated after AESS or dexamethasone treatment (Figure 5I). These inconsistencies may be related to the heterogeneity of the functions of these cytokines in different situations or caused by different modeling methods.

### Inhibiting YAP expression weakened the therapeutic effect of AESS serum on the AD-like cell model

**FIGURE 6:**
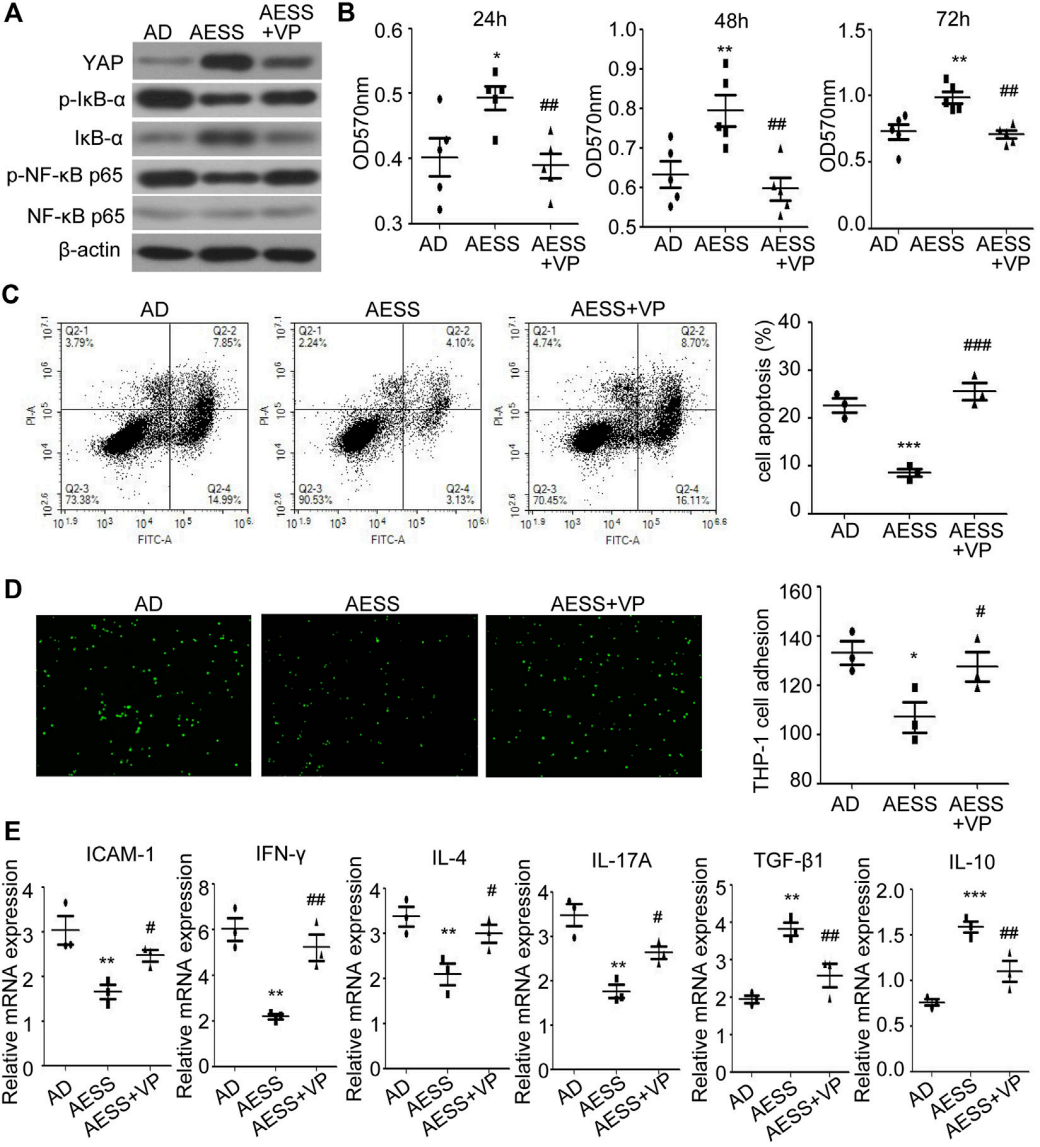
Inhibiting YAP expression could weaken the effect of AESS contained serum on AD-like cell model. **(A)** YAP, IκB-α, p-IκB-α, NF-κB p65 and p-NF-κB p65 expression. **(B)** Cell proliferation was examined by MTT. **(C)** Apoptosis was examined FACS analysis. **(D)** Adhesion to THP-1 cells (100 ×). **(E)** ICAM-1 and inflammatory cytokines (IFN-γ, IL-4, IL-17A, TGF-β and IL-10) mRNA expression. MTT for five replicates. Cell apoptosis, adhesion assay and qRT-PCR for three replicates. AD: AD-like cells were established by HaCaT cells culture with 10 ng/ml TNF-α/IFN-γ; AESS: AD-like cells culture with AESS contained serum; AESS + VP: AD-like cells culture with AESS contained serum and 2 μL YAP inhibitor verteporfin (VP). *p < 0.05, **p < 0.01, ***p < 0.001 *v. s.* AD; #*p* < 0.05, ##*p* < 0.01, ###*p* < 0.001 *v. s.* AESS.

**FIGURE 7:**
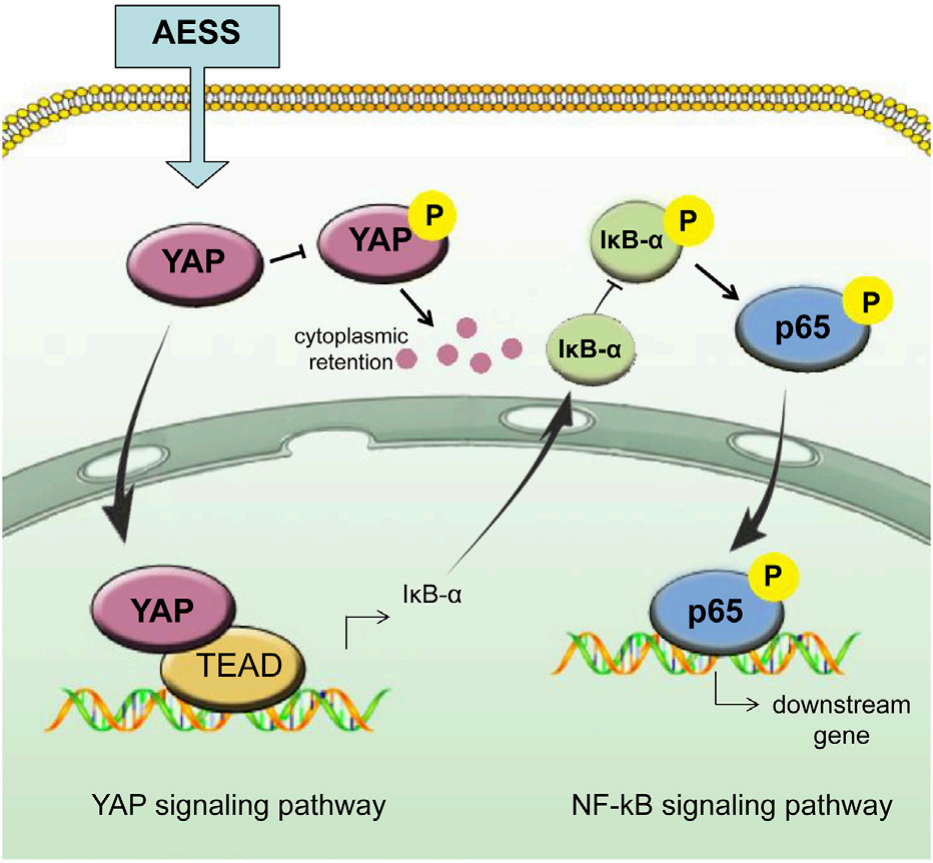
Schematic of AESS’s effect on AD trough the intereaction of YAP and NF-κB signaling pathway.

The YAP inhibitor VP was added to inhibit the expression of YAP in the cells (Figure 6A and Supplemental Figure S1D). Compared with those in the AESS group, the proliferation ability of the cells decreased (Figure 6B), the apoptosis rate increased (Figure 6C), the adhesion rate to the THP-1 cells increased (Figure 6D), and the expression of the p-IκB-α and p-NF-κB p65 proteins recovered (Figure 6A and Supplemental Figure S1D). ICAM-1 and inflammatory cytokine mRNA levels also partially recovered after VP treatment (Figure 6E). This suggests that blocking the expression of YAP partially reduces the curative effect of AESS. The possible mechanism of AESS on AD through the interaction of YAP and the NF-κB signaling pathway is shown in Figure 7.

## Discussion

Disturbance to the immune balance and damage to the skin barrier are recognized as the most important pathogenesis of AD (Langan et al., 2020) but its exact pathogenesis is still unclear. At present, there is still a lack of safe and effective chemical drugs for long-term application, with few side effects, and that avoid recurrence. New biological agents such as dupilumab, a monoclonal antibody targeting IL-4 and IL-13, are still limited in their application owing to their high price and potential risks (Calabrese et al., 2021). Moreover, there is still a big gap in the efficacy of biological agents for the treatment of AD compared with other diseases such as psoriasis (Cline et al., 2019). Since ancient times, TCM has been used in the treatment of diseases in China. It has advantages for chronic recurrent diseases such as AD. Thus, as an important alternative therapy, it has been recognized by many international guidelines. However, the theory of TCM has characteristics of speculation, subjectivity, and fuzziness, which are not conducive to its communication and cooperation around the world. In recent years, many scholars have discussed the molecular biological basis of TCM treatment from macro to micro, subjective to objective, and traditional to modern science and technology, which plays an important role in promoting the scientific and global development of TCM.

The AESS formula is an experienced prescription for AD that was initiated by Dr. Dacan Chen. It has been used in clinical practice for many years (Wu et al., 2014; Liu and Mo, 2019; Jia et al., 2022). However, it is still categorized as empirical medication and the specific pharmacological mechanism is unknown. In this study, animal models and cell models were used to study the specific mechanism of AESS. It was found that AESS can significantly improve the skin lesions of mice, improve the degree of ear swelling, and reduce the dermatitis score, which confirmed its clinical efficacy. Additionally, AESS was found to resolve Th1/Th2/Th17/Treg imbalance in mice and reduce the expression of IgE and IL-17. *In vitro*, AESS reduced the adhesion of HaCaT cells to THP-1 cells, which indicates that AESS plays a role in regulating the immune system in patients with AD.

Previous studies have shown that YAP is highly expressed in the Treg cells of healthy people and mice. The proportion of Th2 and Th17 cells increases and that of Th1 and Treg cells decreases in NC/Nga mice induced by DNFB, and the expression of YAP is increased in Th17 cells and decreased in Treg cells (Jia et al., 2021), which was different from the results of this study. In DNFB-stimulated Balb/C mice, the proportion of Th1 cells decreased, the proportion of Th2, Th17, and Treg cells increased, and the expression of YAP increased in both Th17 and Treg cells. The difference in the Treg cell proportion and YAP expression in Treg cells may be related to the different mouse breeds (Nc/Nga mice or Balb/C mice) and topical stimulants (DNFB or dinitrochlorobenzene [DNCB]). Previous studies have reported that the number and function of Treg cells in AD are different; some reported that the decrease in Treg cell number and inhibition of Treg cell function promote the inflammatory response in AD (Ma et al., 2014) but there are also reports about increases (Roesner et al., 2015) or even no significant change (Hayashida et al., 2011). Consequently, the role of Treg cells in the pathogenesis of AD still needs more research to reach a consensus. However, it is certain that increasing the expression of YAP in the initial CD4^+^ T cells and then inducing differentiation into Th17 or Treg cells in different directions will increase the proportion of Th17 or Treg cells. However, inhibiting the expression of YAP will decrease the proportion of the initial CD4^+^ T cells differentiating into Th17 or Treg cells. This indicates that the change in YAP expression affects the differentiation of Th17 and Treg cells, thereby causing Th17/Treg imbalance in AD (Jia et al., 2021). Therefore, as the change in YAP expression causes Th17/Treg imbalance, AESS can resolve this to a certain extent. The ratio of Th1/Th2/Th17/Treg cells decreased significantly after dexamethasone treatment, which indicates that glucocorticoids have a broad-spectrum and strong immunosuppressive effect but the side effects should be noted.

Th17 cells play an important role in skin inflammation in AD. Th17 cells and serum IL-17 levels are elevated in patients with AD compared with healthy controls (Mao et al., 2021). Currently, IL-17 is considered to participate in the acute phases of AD (Koga et al., 2008; Jia et al., 2021). IL-17 promotes neutrophil recruitment, activation, and aggregation; monocyte and macrophage infiltration into wounded skin; and scar formation. Immunohistochemically, IL-17 + cells infiltrate the papillary dermis of AD lesioned skin. IL-17 secretion can be enhanced by the *Staphylococcus aureus*-derived superantigen staphylococcal enterotoxin B (Sugaya, 2020). IL-17 triggers abnormal keratinocyte proliferation and parakeratosis, suggesting its potential role in epidermal barrier dysfunction in AD (Wongvibulsin et al., 2021). Our study showed that AESS can inhibit Th17 cell responses (both Th17 cells and serum IL-17 levels) in an AD mouse model, inhibit keratinocyte apoptosis, and inhibit adhesion to monocytes and inflammatory cytokines (including IL-17) in an AD cell model, indicating that AESS plays a role in both acute phase inflammation and the skin barrier *via* the Th17 cell response in AD.

We studied the specific target and pathway mechanism of AESS using cell and animal models. In mouse skin, YAP expression decreased in the AD group, which is consistent with previous research (Jia et al., 2021). Notably, the integrity of skin structure is very important as a good skin barrier and the *epidermis* is mainly composed of keratinocytes (Egawa and Kabashima, 2016). The decreased expression of YAP in AD keratinocytes may damage the barrier function of the *epidermis* by reducing its proliferation ability and hindering differentiation, leading to the clinical phenomenon of skin erosion and difficulty healing. The AESS formula partly restored the expression of YAP in animal and cell models. Moreover, the proliferation ability of the AD-like inflammatory cell model cultured with AESS serum increased and the apoptosis rate decreased. This effect was decreased after inhibiting the expression of YAP, which indicates that AESS plays an important role in restoring the function of keratinocytes and the skin barrier by increasing the expression of YAP. IκB-α and NF-κB subunits are inactivated in the cytoplasm at rest. When IκB kinase (IKK) is activated by upstream signaling, the activated IKK will ubiquitinate, phosphorylate, and degrade IκB-α, which will activate the two subunits of NF-κB from the inactivated state, phosphorylate them, and transfer them from the cytoplasm to the nucleus (especially the p65 subunits). Then, they will bind with the corresponding inflammation-related genes, start the transcription of inflammatory cytokines, and induce inflammation (Chen et al., 2020). Our results showed that the expression of phosphorylated IκB-α and NF-κB p65 increased in the AD-like inflammatory cell model, indicating that the activity of the NF-κB signaling pathway increased. The addition of AESS serum culture inhibited the expression of phosphorylated IκB-α and NF-κB p65, which means that its therapeutic effect is achieved by inhibiting the activity of the NF-κB signaling pathway.

The Hippo-YAP pathway interacts with many other signal pathways in a very complex cellular signal transmission network. The crosstalk between Hippo-YAP and NF-κB signaling pathways also seems complex. Qiu et al. (2022) showed that melatonin-mediated YAP upregulation attenuates TNF-α–induced NF-κB pathway activation by enhancing the expression of IκBα in nucleus pulposus cells. YAP also inhibits the activation of NF-κB signaling prompted by TNF-α in murine preosteoblastic cells (Yang et al., 2020) and cementoblast cells (Zhang et al., 2020). These studies indicate that Hippo-YAP signaling may be upstream and negatively correlated with the activity of NF-κB signaling. Our study showed that YAP was downregulated and NF-κB signaling activity was upregulated in AD-like keratinocytes, and these trends can be mitigated by AESS treatment. Thus, we infer that AESS plays its role by upregulating YAP and inhibiting NF-κB signaling activity in AD (Figure 7). However, another study has shown that inhibiting the NF-κB pathway rescues the reduction of YAP expression under titanium ion exposure (Tang et al., 2021). The crosstalk between Hippo-YAP and NF-κB pathways may be intricate depending on different cells and intracellular conditions, and more research is needed to clarify the specific pathways in AD.

In conclusion, we found evidence of the mechanisms of AESS in AD, which can be used to promote its use as an alternative medication for prolonged use with fewer side effects. However, the dose of animal study on the compound prescription of traditional chinese medicine is generally based on the conversion of commonly clinical dose of human. The metabolism of rodents is different from that of humans, which may bring some limitations. The effect of AESS on epidermal barrier function and the specific signaling pathway and mechanism still need more research.

## Data Availability

The original contributions presented in the study are included in the article/Supplementary Materials, further inquiries can be directed to the corresponding authors.
