# Supplementary material for: Therapeutic effect of chinese herbal medicine gu-ben-hua-shi (AESS) formula on atopic dermatitis through regulation of yes-associated protein
Source: Front Pharmacol. 2022 Oct 12;13:929580. doi: 10.3389/fphar.2022.929580 (PMC9597468; doi:10.3389/fphar.2022.929580)
Supplement: Supplementary file 1 [file DataSheet1.docx]

Supplementary Material

# Supplementary Figures and Tables

## Supplementary Figures


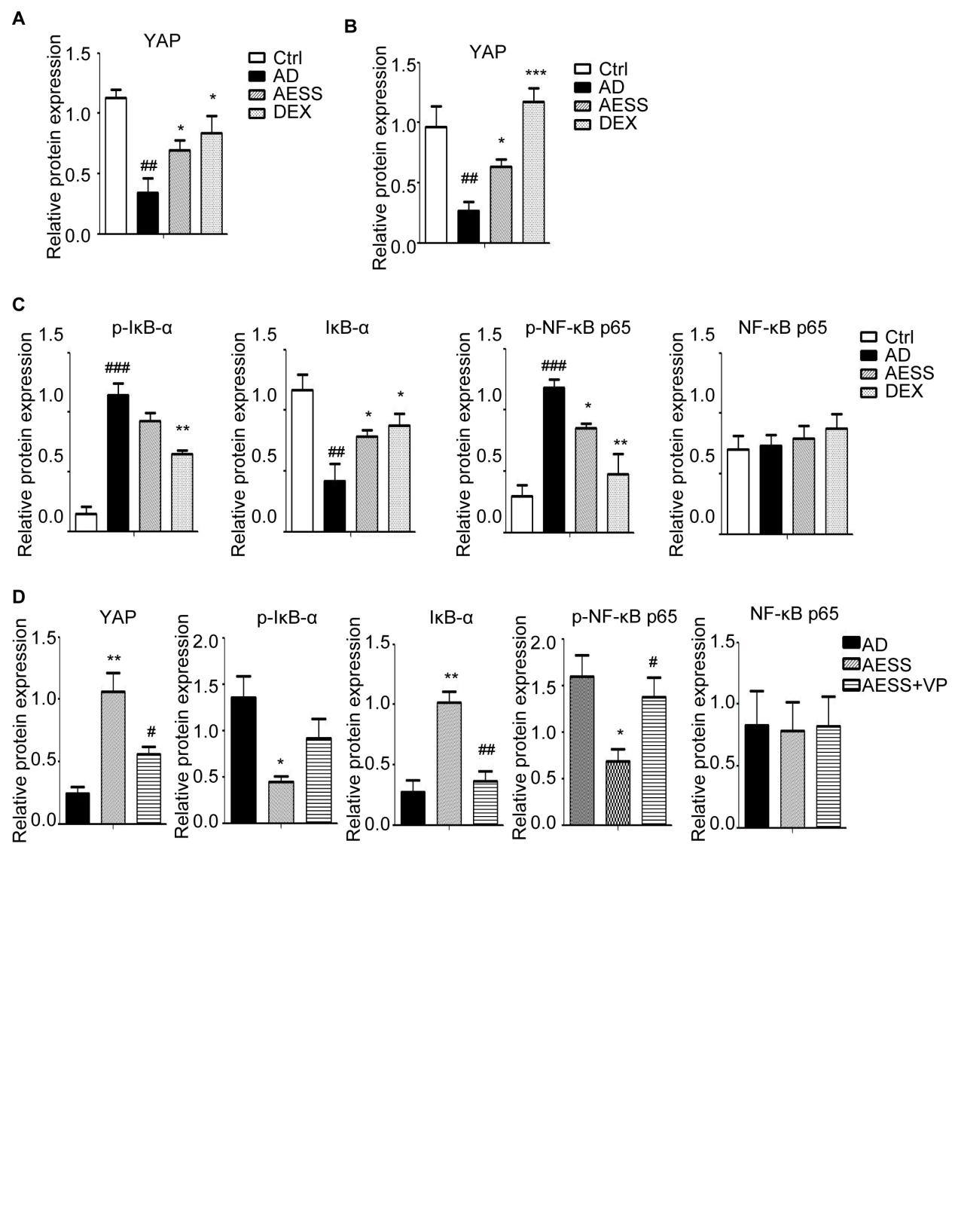


**Supplementary Figure 1.** **Relative protein expression of Western blot analysis. (A)** Relative protein expression in Figure 4C. **(B)** Relative protein expression in Figure 5G. **(C)** Relative protein expression in Figure 5H. **(D)** Relative protein expression in Figure 6A. Western Blot for 3 replicates. Ctrl: Control mice or HaCaT cells; AD: AD-like mice or cells; AESS: mice or cells treated with AESS; DEX: mice or cells treated with dexamethasone. AESS+VP: AD-like cells culture with AESS contained serum and 2μL YAP inhibitor verteporfin (VP). #*P*<0.05, ##*P*<0.01, ###*P*<0.001 v.s. Ctrl in (A)(B)(C) or AESS in (D); **P*<0.05, ***P*<0.01, ****P*<0.001 v.s. AD.

## Supplementary Tables

Supplemental Table S1. The information of three batches of single herbs

| Batch | Chinese name | Batch number | Place of origin |
| --- | --- | --- | --- |
| S1 | fangfeng | 2007001 | Heilongjiang Province |
|  | ezhu | 191203761 | Guangxi province |
|  | jinyinhua | 1911002 | Shandong province |
|  | shengdihuang | 191000039 | Henan province |
|  | huaihua | 200901 | Jiangsu province |
|  | baizhu | 1810002 | Zhejiang province |
|  | yiyiren | 181105921 | Guizhou province |
|  | cangzhu | 191004471 | Inner Mongolia Autonomous Region |
| S2 | fangfeng | 2006001 | Heilongjiang Province |
|  | ezhu | 201101 | Guangxi province |
|  | jinyinhua | 2008001 | Shandong province |
|  | shengdihuang | 191000039 | Henan province |
|  | huaihua | 190504381 | Liaoning province |
|  | baizhu | 2101001 | Zhejiang province |
|  | yiyiren | 2011001 | Guizhou province |
|  | cangzhu | YPBOH0002 | Inner Mongolia Autonomous Region |
| S3 | fangfeng | 2105001 | Heilongjiang Province |
|  | ezhu | 170813451 | Guangxi province |
|  | jinyinhua | 2012002 | Shandong province |
|  | shengdihuang | 210401 | Guangzhou |
|  | huaihua | 2012001 | Jiangsu province |
|  | baizhu | YPA9K0001 | Zhejiang province |
|  | yiyiren | 2003001 | Guizhou province |
|  | cangzhu | 210401 | Hebei province |

Supplemental Table S2. Mobile phase system of UPLC

| Time (min) | Acetonitrile (%) | 0.1% Formic acid (%) |
| --- | --- | --- |
| 0 | 7 | 93 |
| 2 | 7 | 93 |
| 5 | 15 | 85 |
| 10 | 15 | 85 |
| 14 | 20 | 80 |
| 18 | 20 | 80 |
| 20 | 7 | 93 |

Supplemental Table S3. The qRT-PCR primers used in this study

| Gene name | Sequences |
| --- | --- |
| mouse-YAP | Forward: 5’-GACAGTGCTCTCTGACCTTATC-3’ |
|  | Reverse: 5’-GCTGGACACAACAAGAAAGAC-3’ |
| mouse-GAPDH | Forward: 5’-GGCCTCCAAGGAGTAAGAAA-3’ |
|  | Reverse: 5’-GCCCCTCCTGTTATTATGG-3’ |
| human-ICAM-1 | Forward: 5’-GCAAGAAGATAGCCAACCAA-3’ |
|  | Reverse: 5’-TGCCAGTTCCACCCGTTC-3’ |
| human-IFN-γ | Forward: 5’-ATTCAGATGTAGCGGATAA-3’ |
|  | Reverse: 5’-TGTATTGCTTTGCGTTG-3’ |
| human-IL-4 | Forward: 5’-CCCTCTGTTCTTCCTGC-3’ |
|  | Reverse: 5’-TACGGTCAACTCGGTGC-3’ |
| human-IL-17A | Forward: 5’-CACTGCTACTGCTGCTGA -3’ |
|  | Reverse: 5’-TGAGGTGGATCGGTTGT-3’ |
| human-TGF-β1 | Forward: 5’-AACCCACAACGAAATCTATGAC-3’ |
|  | Reverse: 5’-GCTGAGGTATCGCCAGGAAT-3’ |
| human-IL-10 | Forward: 5’-ACCAAGACCCAGACATCA-3’ |
|  | Reverse: 5’-TTCACAGGGAAGAAATCG-3’ |
| human-β-actin | Forward: 5’-GGCACCCAGCACAATGAA-3’ |
|  | Reverse: 5’-TAGAAGCATTTGCGGTGG-3’ |

Supplemental Table S4. Mobile phase system of UPLC-MS/MS

| Time (min) | Acetonitrile (%) | 0.1% Formic acid (%) |
| --- | --- | --- |
| 0 | 5 | 95 |
| 2 | 5 | 95 |
| 24 | 54.5 | 45.5 |
| 25 | 54.5 | 45.5 |
| 25.1 | 5 | 95 |
| 28 | 5 | 95 |

Supplemental Table S5. The declustering potential and collision energy of compounds

| Compound | Ion(m/z) | MS/MS | DP(V) | CE(V) |
| --- | --- | --- | --- | --- |
| Neochlorogenic acid | 353.1 | 191.0 | -73 | -27 |
|  |  | 135.1 | -59 | -25 |
|  |  | 179.1 | -40 | -37 |
| Chlorogenic acid | 353.0 | 191.0 | -77 | -20 |
| Cryptochlorogenic acid | 353.0 | 191.3 | -75 | -24 |
|  |  | 135.2 | -25 | -38 |
|  |  | 179.1 | -48 | -21 |
|  |  | 173.1 | -40 | -20 |
| Prim-*O*-glucosylcimifugin | 469.4 | 307.0 | +171 | +39 |
| Rutin | 609.0 | 300.2 | -134 | -52 |
|  |  | 271.0 | -80 | -88 |
| Kaempferol-3-*O*-Rutinoside | 593.1 | 285.2 | -182 | -39 |
| Narcissoside | 623.0 | 315.2 | -160 | -43 |
|  |  | 300.0 | -104 | -58 |
| Isochlorogenic acid A | 515.0 | 191.0 | -26 | -41 |
|  |  | 178.9 | -43 | -40 |
|  |  | 353.1 | -16 | -21 |
|  |  | 135.1 | -52 | -60 |
| 5-*O*-methylvisamitol glycoside | 497.1 | 270.8 | -73 | -26 |
| Isochlorogenic acid C | 515.1 | 172.9 | -79 | -42 |
|  |  | 179.0 | -57 | -39 |
|  |  | 191.0 | -68 | -51 |
|  |  | 353.1 | -48 | -26 |
|  |  | 135.1 | -76 | -61 |
